# The Oscillation on Solutions of Some Classes of Linear Differential Equations with Meromorphic Coefficients of Finite [*p*, *q*]-Order

**DOI:** 10.1155/2013/243873

**Published:** 2013-12-16

**Authors:** Hong-Yan Xu, Jin Tu, Zu-Xing Xuan

**Affiliations:** ^1^Department of Informatics and Engineering, Jingdezhen Ceramic Institute, Jingdezhen, Jiangxi 333403, China; ^2^Institute of Mathematics and Informatics, Jiangxi Normal University, Nanchang, Jiangxi 330022, China; ^3^Beijing Key Laboratory of Information Service Engineering, Department of General Education, Beijing Union University, No. 97 Bei Si Huan Dong Road, Chaoyang District, Beijing 100101, China

## Abstract

This paper considers the oscillation on meromorphic solutions of the second-order linear differential equations with the form *f*′′ + *A*(*z*)*f* = 0, where *A*(*z*) is a meromorphic function with [*p*, *q*]-order. We obtain some theorems which are the improvement and generalization of the results given by Bank and Laine, Cao and Li, Kinnunen, and others.

## 1. Introduction and Main Results

The purpose of this paper is to study the oscillation on solutions of linear differential equations in the complex plane. It is well known that Nevanlinna theory has appeared to be a powerful tool in the field of complex differential equations. We assume that readers are familiar with the standard notations and the fundamental results of the Nevanlinna's value distribution theory of meromorphic functions (see [1, 3, 4]). Throughout the paper, a meromorphic function *f* means meromorphic in the complex plane *ℂ*. In addition, we use *σ*(*f*) and *λ*(*f*) to denote the order and the exponent of convergence of zero sequence of meromorphic function *f*(*z*), respectively. For sufficiently large *r* ∈ [1, *∞*), we define log⁡⁡_*i*+1_ *r* = log⁡⁡_*i*_(log⁡⁡*r*)  (*i* ∈ *N*) and exp⁡_*i*+1_ *r* = exp⁡(exp⁡_*i*_ *r*)  (*i* ∈ *N*) and exp⁡_0_ *r* = *r* = log⁡⁡_0_ *r*, exp⁡_−1_ *r* = log⁡⁡*r*.

For the second-order linear differential equation,

(1) $$\begin{array}{c} f^{\prime \prime }+A\left(z\right)f=0, \end{array}$$

where *A*(*z*) is an entire function or meromorphic function of finite order. In 1982, Bank and Laine [5] mainly studied the distribution of zeros of solutions of (1) when *A* is an entire function of finite order.

Obviously, all solutions of (1) are entire when *A*(*z*) is entire. However, there are some immediate difficulties when *A* is meromorphic; for example, the solutions of (1) may not be entire, and it is possible that no solution of (1) except the zero solution is single-valued on the plane. In 1983, Bank and Laine [6] investigated the exponent of convergence of zero sequence of nontrivial solutions of (1), when *A* is meromorphic function and obtained the results as follows.

**Theorem 1 (see [6, Theorem 5])**

Let *A* be a transcendental meromorphic function of order *σ*(*A*), where 0 < *σ*(*A*) ≤ *∞*. Assume that $\bar{\lambda }(A)<\sigma (A)$. Then, if *f*≢0 is a meromorphic solution of (1), one has

(2) $$\begin{array}{c} \sigma \left(A\right)\leq \max\left\{\bar{\lambda }\left(f\right),\bar{\lambda }\left(\frac{1}{f}\right)\right\}. \end{array}$$

**Theorem 2 (see [6, Theorem 6])**

Let *A* be a transcendental meromorphic function. Assume that (1) possesses two linearly independent meromorphic solutions *f*_1_ and *f*_2_ satisfying $\bar{\lambda }(f_{1})<\infty$, $\bar{\lambda }(f_{2})<\infty$. Then, any solution *f*≢0 of (1) which is not a constant multiple of either *f*_1_ or *f*_2_ satisfies

(3) $$\begin{array}{c} \max\left\{\bar{\lambda }\left(f\right),\bar{\lambda }\left(\frac{1}{f}\right)\right\}=\infty , \end{array}$$

unless all solutions of (1) are of finite order. In the special case where $\bar{\lambda }\left(1/A\right)<\infty$, one can conclude that $\bar{\lambda }(f)=\infty$ unless all solutions of (1) are of finite order.

After their work, many authors have investigated the growth and the exponent of convergence of zero sequence of non-trivial solutions of (1) and obtained many classical results (see [5, 7, 8]).

In 1998, Kinnunen [9] further investigated the oscillation results of entire solutions of (1) when *A*(*z*) is an entire function with finite iterated order and obtained some theorems which improved some theorems given by Bank and Laine [5]. Later, Chen [10] and Wang and Lü [11] studied the oscillation of solutions of (1) when *A*(*z*) is a meromorphic function with finite order by using the Wiman-Valiron theory; they obtained some results which extend some theorems of Kinnunen [9]. In 2007, Liang and Liu [12] considered the complex oscillation on (1) when *A*(*z*) is a meromorphic function with many finite poles. They extended the above oscillation results by using the method of the Wiman-Valiron theory. Although the Wiman-Valiron theory is a powerful tool to investigate entire solutions, it is only useful for the meromorphic function *A*(*z*) with the exponent of the convergence sequence of poles which is less than the order of *A*(*z*) if we consider (1). In 2010, Cao and Li [13] made use of a result due to Chiang and Hayman [14] instead of the Wiman-Valiron theory and obtained four oscillation theorems and three corollaries which extended the above results due to Bank-Laine, Kinnunen, and Liang and Liu.

**Theorem 3 (see [13, Theorem 1.6])**

Let *A*(*z*) be a meromorphic function with 0 < *i*(*A*) = *n* < *∞*, and assume that ${\bar{\lambda }}_{n}(A)<\sigma_{n}(A)\neq 0$. Then, if *f* is a nonzero meromorphic solution of (1), one has $\sigma_{n}\left(A\right)\leq \max\left\{{\bar{\lambda }}_{n}\left(f\right),{\bar{\lambda }}_{n}\left(1/f\right)\right\}$. In the special case where either *δ*(*∞*, *f*) > 0 or the poles of *f* are of uniformly bounded multiplicities, one can conclude that $\max\left\{\lambda_{n+1}(f),\lambda_{n+1}\left(1/f\right)\right\}\leq \sigma_{n+1}(A)\leq \max\left\{{\bar{\lambda }}_{n}(f),{\bar{\lambda }}_{n}\left(1/f\right)\right\}$.

**Theorem 4 (see [13, Theorem 1.7])**

Let *A* be a meromorphic function with 0 < *i*(*A*) = *n* < *∞*. Assume that (1) possesses two linearly independent meromorphic solutions *f*_1_ and *f*_2_. Denote *E*∶ = *f*_1_ *f*_2_. If ${\bar{\lambda }}_{n}(E)<\infty$, then any nonzero solution *f* of (1) which is not a constant multiple of either *f*_1_ or *f*_2_ satisfies ${\bar{\lambda }}_{n}(f)=\infty$, unless all solutions of (1) are of finite iterated *n*-order. In the special case where *δ*(*∞*, *A*) > 0  *i*_*λ*_(1/*A*) < *n*, or *λ*_*n*_(1/*A*) < *σ*_*n*_(*A*) (e.g., *A* is an entire function), one can conclude that ${\bar{\lambda }}_{n}=\infty$.

**Theorem 5 (see [13, Theorem 1.9])**

Let *A* be a meromorphic function with 0 < *i*(*A*) = *n* < *∞*. Assume that *f*_1_ and *f*_2_ are two linearly independent meromorphic solutions of (1) such that max⁡⁡{*λ*_*n*_(*f*_1_), *λ*_*n*_(*f*_2_)} < *σ*_*n*_(*A*). Let Π(*z*)≢0 be any meromorphic function for which *σ*_*n*_(Π) < *σ*_*n*_(*A*). Let *g*_1_ and *g*_2_ be two linearly independent solutions of the differential equation *g*′′ + (*A*(*z*) + Π(*z*))*g* = 0. Denote *E*∶ = *f*_1_ *f*_2_ and *F*∶ = *g*_1_ *g*_2_. If

(4) max⁡⁡{iλ(1E),iλ(1F)}<n  or max⁡{λn(1E),λn(1F)}<λn(A),

then max⁡⁡{*λ*_*n*_(*g*_1_), *λ*_*n*_(*g*_2_)} ≥ *σ*_*n*_(*A*).

In 1976, Juneja and his coauthors [15, 16] firstly introduced the concept of [*p*, *q*]-order of entire functions. Recently, Belaïdi [17] and Liu et al. [18] investigated the growth of solutions of complex differential equations

(5) $$\begin{array}{c} f^{\left(n\right)}+A_{n-1}\left(z\right)f^{\left(n-1\right)}+\cdots +A_{1}\left(z\right)f^{\prime }+A_{0}\left(z\right)f=0, \end{array}$$

where *A*_*j*_(*z*), (*j* = 0,1,…, *n* − 1) are entire or meromorphic functions with finite [*p*, *q*]-order, by using the idea of [*p*, *q*]-order, and obtained some interest results which improved and extended some previous theorems given by [5–7, 9, 19].

Thus, it is interesting to consider the complex oscillation on the meromorphic solutions of (1) for the case when *A* is entire or meromorphic functions in the terms of the idea of [*p*, *q*]-order.

In this paper, we further investigated the complex oscillation of meromorphic solutions of (1) when *A*(*z*) is meromorphic by using the idea of [*p*, *q*]-order. To state our theorems, we first introduce the concepts of entire functions of [*p*, *q*]-order (see [15, 16, 18]). Throughout this paper, we always assume that *p*, *q* are positive integers satisfying *p* ≥ *q* ≥ 1.

**Definition 6**

If *f*(*z*) is a transcendental entire function, the [*p*, *q*]-order of *f*(*z*) is defined by

(6) $$\begin{array}{c} \sigma_{\left[p,q\right]}\left(f\right)=\underset{r\to \infty }{\mathrm{limsup}}\frac{\log_{p+1}M\left(r,f\right)}{\log_{q}r}=\underset{r\to \infty }{\mathrm{limsup}}\frac{\log_{p}T\left(r,f\right)}{\log_{q}r}. \end{array}$$

**Remark 7**

If *f*(*z*) is a polynomial, then *σ*_[*p*,*q*]_(*f*) = 0 for any *p* ≥ *q* ≥ 1. By Definition 6, we have that *σ*_[1,1]_(*f*) = *σ*_1_(*f*) = *σ*(*f*), *σ*_[*p*+1,1]_(*f*) = *σ*_*p*_(*f*).

**Remark 8**

If *f*(*z*) is an entire function satisfying 0 < *σ*_[*p*,*q*]_(*f*) < *∞*, then

- *σ*_[*p*−*n*,*q*]_(*f*) = *∞*(*n* < *p*), *σ*_[*p*,*q*−*n*]_(*f*) = 0(*n* < *q*), *σ*_[*p*+*n*,*q*+*n*]_(*f*) = 1(*n* < *p*) for *n* = 1,2,….
- if [*p*′, *q*′] is any pair of integers satisfying *q*′ = *p*′ + *q* − *p* and *p*′ < *p*, then *σ*_[*p*′,*q*′]_(*f*) = 0 if 0 < *σ*_[*p*,*q*]_(*f*) < 1 and *σ*_[*p*′,*q*′]_(*f*) = *∞* if 1 < *σ*_[*p*,*q*]_(*f*) < *∞*;
- *ρ*_[*p*′,*q*′]_(*f*) = *∞* for *q*′ − *p*′ > *q* − *p* and *ρ*_[*p*′,*q*′]_(*f*) = 0 for *q*′ − *p*′ < *q* − *p*.

**Definition 9**

A transcendental meromorphic function *f*(*z*) is said to have index-pair [*p*, *q*] if 0 < *σ*_[*p*,*q*]_(*f*) < *∞* and *σ*_[*p*−1,*q*−1]_(*f*) is not a nonzero finite number.

**Definition 10**

Let *f*_1_, *f*_2_ be two entire functions such that *σ*_[*p*_1_,*q*_1_]_(*f*_1_) = *σ*_1_, *σ*_[*p*_2_,*q*_2_]_(*f*_2_) = *σ*_2_ and *p*_1_ ≤ *p*_2_. Then the following results about their comparative growth can be easily deduced.

- If *p*_2_ − *p*_1_ > *q*_2_ − *q*_1_, then the growth of *f*_1_ is slower than the growth of *f*_2_;
- If *p*_2_ − *p*_1_ < *q*_2_ − *q*_1_, then *f*_1_ grows faster than *f*_2_.
- If *p*_2_ − *p*_1_ = *q*_2_ − *q*_1_ > 0, then the growth of *f*_1_ is slower than the growth of *f*_2_ if *σ*_2_ ≥ 1 while the growth of *f*_1_ is faster than the growth of *f*_2_ if *ρ*_2_ < 1.
- Let *p*_2_ − *p*_1_ = *q*_2_ − *q*_1_ = 0; then *f*_1_, *f*_2_ are of the same index-pair [*p*_1_, *q*_1_]. If *σ*_1_ > *σ*_2_, then *f*_1_ grows faster than *f*_2_, and if *σ*_1_ < *σ*_2_, then *f*_1_ grows slower than *f*_2_. If *σ*_1_ = *σ*_2_, Definition 6 does not give any precise estimate about the relative growth of *f*_1_ and *f*_2_.

**Definition 11 (see [15, 16, 18])**

The [*p*, *q*] exponent of convergence of the zero sequence and the [*p*, *q*] exponent of convergence of the distinct zero sequence of *f*(*z*) are defined respectively, by

(7) $$\begin{array}{c} \lambda_{\left[p,q\right]}\left(f\right)=\bar{\underset{r\to \infty }{\lim}}\frac{\log_{p}n\left(r,1/f\right)}{\log_{q}r}=\bar{\underset{r\to \infty }{\lim}}\frac{\log_{p}N\left(r,1/f\right)}{\log_{q}r},\\ {\bar{\lambda }}_{\left[p,q\right]}\left(f\right)=\bar{\underset{r\to \infty }{\lim}}\frac{\log_{p}\bar{n}\left(r,1/f\right)}{\log_{q}r}=\bar{\underset{r\to \infty }{\lim}}\frac{\log_{p}\bar{N}\left(r,1/f\right)}{\log_{q}r}. \end{array}$$

**Remark 12**

It is easy to know that

(8) $$\begin{array}{c} {\bar{\lambda }}_{\left[p,q\right]}\left(f\right)\leq \lambda_{\left[p,q\right]}\left(f\right)\leq \sigma_{\left[p,q\right]}\left(f\right). \end{array}$$

Now, we will show our main results on the complex oscillation on meromorphic solutions of (1) when *A*(*z*) is meromorphic with finite [*p*, *q*]-order as follows.

**Theorem 13**

Let *A*(*z*) be a transcendental meromorphic function with *σ*_[*p*,*q*]_(*A*) > 0. Assume that ${\bar{\lambda }}_{\left[p,q\right]}(A)<\sigma_{\left[p,q\right]}(A)$. Then, if *f* is a nonzero meromorphic solution of (1), one has

(9) $$\begin{array}{c} \sigma_{\left[p,q\right]}\left(A\right)\leq \max\left\{{\bar{\lambda }}_{\left[p,q\right]}\left(f\right),{\bar{\lambda }}_{\left[p,q\right]}\left(\frac{1}{f}\right)\right\}. \end{array}$$

In the special case where either *δ*(*∞*, *f*) > 0 or the poles of *f* are of uniformly bounded multiplicities, one can get that

(10) max⁡⁡{λ[p+1,q](f),λ[p+1,q](1f)}    ≤σ[p+1,q](A)  ≤max⁡⁡{λ¯[p,q](f),λ¯[p,q](1f)}.

**Theorem 14**

Let *A*(*z*) be a transcendental meromorphic function with *σ*_[*p*,*q*]_(*A*)∶ = *σ*(0 < *σ* < +*∞*). Assume that (1) possesses two linearly independent meromorphic solutions *f*_1_ and *f*_2_. Denote *E*∶ = *f*_1_ *f*_2_. If ${\bar{\lambda }}_{\left[p,q\right]}(E)<\infty$, then any nonzero solution *f* of (1) which is not a constant multiple of either *f*_1_ or *f*_2_ satisfies ${\bar{\lambda }}_{\left[p,q\right]}(f)=\infty$, unless all solutions of (1) are of finite [*p*, *q*]-order. In the special case where *δ*(*∞*, *A*) > 0 or *λ*_[*p*,*q*]_(1/*A*) < *σ*_[*p*,*q*]_(*A*), one can conclude that ${\bar{\lambda }}_{\left[p,q\right]}=\infty$.

**Theorem**

Let *A*(*z*) be a transcendental meromorphic function with *σ*_[*p*,*q*]_(*A*) > 0. Assume that (1) possesses two linearly independent meromorphic solutions *f*_1_ and *f*_2_. Denote *E*∶ = *f*_1_ *f*_2_. If *δ*(*∞*, *A*) > 0 or ${\bar{\lambda }}_{\left[p,q\right]}\left(1/A\right)<\sigma_{\left[p,q\right]}(A)$, and if either *δ*(*∞*, *f*) > 0 or the poles of *f* are of uniformly bounded multiplicities, then one has

(11) λ¯[p+1,q](E)=λ[p+1,q](E)=σ[p+1,q](E)=max⁡⁡{λ¯[p+1,q](f1),λ¯[p+1,q](f2)}≤λ[p+1,q](f1)=λ[p+1,q](f2)=λ[p,q](A).

**Theorem 16**

Let *A* be a meromorphic function with *σ*_[*p*,*q*]_(*A*) > 0. Assume that *f*_1_ and *f*_2_ are two linearly independent meromorphic solutions of (1) such that

(12) $$\begin{array}{c} \max\left\{\lambda_{\left[p,q\right]}\left(f_{1}\right),\lambda_{\left[p,q\right]}\left(f_{2}\right)\right\}<\sigma_{\left[p,q\right]}\left(A\right). \end{array}$$

Let Π(*z*)≢0 be any meromorphic function for which *σ*_[*p*,*q*]_(Π) < *σ*_[*p*,*q*]_(*A*). Let *g*_1_ and *g*_2_ be two linearly independent solutions of the differential equation

(13) $$\begin{array}{c} g^{\prime \prime }+\left(A\left(z\right)+\Pi \left(z\right)\right)g=0. \end{array}$$

Denote *E*∶ = *f*_1_ *f*_2_ and *F*∶ = *g*_1_ *g*_2_. If

(14) $$\begin{array}{c} \max\left\{\lambda_{\left[p,q\right]}\left(\frac{1}{E}\right),\lambda_{\left[p,q\right]}\left(\frac{1}{F}\right)\right\}<\lambda_{\left[p,q\right]}\left(A\right), \end{array}$$

then max⁡⁡{*λ*_[*p*,*q*]_(*g*_1_), *λ*_[*p*,*q*]_(*g*_2_)} ≥ *σ*_[*p*,*q*]_(*A*).

**Remark 17 (Following Hayman [20])**

we will use the abbreviation “n.e.” (nearly everywhere) to mean “everywhere in (0, +*∞*) except in a set of finite measure” in the proofs of our main results of this paper.

**Remark 18**

Obviously, Theorems 13–16 are the improvement of Theorems 3–5 given by Cao and Li [13].

## 2. Some Lemmas

For the proof of our results we need the following lemmas.

**Lemma 19 (see [20, Theorem 4])**

Let *f*(*z*) be a transcendental meromorphic function not of the form *e* ^*αz*+*β*^. Then

(15) T(r,ff′)≤3N¯(r,f)+7N¯(r,1f) +4N¯(r,1f′′)+S(r,ff′).

Using the same proof of Remark  1.3 in [9], one can easily prove the following lemma.

**Lemma 20**

Let *f*(*z*) be a meromorphic function of [*p*, *q*]-order. Then

(16) $$\begin{array}{c} \sigma_{\left[p,q\right]}\left(f\right)=\sigma_{\left[p,q\right]}\left(f^{\prime }\right). \end{array}$$

**Lemma 21**

Let *f*(*z*) be a meromorphic function with [*p*, *q*]-order and *σ*_[*p*,*q*]_(*f*) = *σ*, and let *k* ≥ 1 be an integer. Then for any *ε* > 0,

(17) $$\begin{array}{c} m\left(r,\frac{f^{\left(k\right)}}{f}\right)=O\left\{\exp_{p-1}\left\{\left(\sigma +\varepsilon \right)\log_{q}r\right\}\right\} \end{array}$$

holds outside of an exceptional set *E*_1_ of finite linear measure.

**Proof**

Let *k* ≥ 1. Since *σ* = *σ*_[*p*,*q*]_(*f*) < *∞*, we have for all sufficiently large *r*

(18) $$\begin{array}{c} T\left(r,f\right)<\exp_{p}\left\{\left(\sigma +\varepsilon \right)\log_{q}r\right\}. \end{array}$$

By the lemma of the logarithmic derivative, we have

(19) $$\begin{array}{c} m\left(r,\frac{f^{\left(k\right)}}{f}\right)=O\left\{\log T\left(r,f\right)+\log r\right\},\;\left(r\notin E_{1}\right), \end{array}$$

where *E*_1_ ⊂ (1, *∞*) is a set of finite linear measure, not necessarily the same at each occurrence. Hence we have

(20) $$\begin{array}{c} m\left(r,\frac{f^{\prime }}{f}\right)=O\left\{\exp_{p-1}\left\{\left(\sigma +\varepsilon \right)\log_{q}r\right\}\right\},\;\left(r\notin E_{1}\right). \end{array}$$

Next, assume that we have

(21) $$\begin{array}{c} m\left(r,\frac{f^{\left(k\right)}}{f}\right)=O\left\{\exp_{p-1}\left\{\left(\sigma +\varepsilon \right)\log_{q}r\right\}\right\},\;\left(r\notin E_{1}\right), \end{array}$$

for some *k* ∈ *N*. Since *N*(*r*, *f* ^(*k*)^)≤(*k* + 1)*N*(*r*, *f*), it holds that

(22) T(r,f(k)) ≤m(r,f(k))+N(r,f(k)) ≤m(r,f(k)f)+m(r,f)+(k+1)N(r,f) ≤(k+1)T(r,f)+O{exp⁡p−1{(σ+ε)log⁡qr}},(r∉E1).

By (20), we again obtain

(23) $$\begin{array}{c} m\left(r,\frac{f^{\left(k+1\right)}}{f^{\left(k\right)}}\right)=O\left\{\exp_{p-1}\left\{\left(\sigma +\varepsilon \right)\log_{q}r\right\}\right\},\;\left(r\notin E_{1}\right), \end{array}$$

and hence, for sufficiently large *r* ∉ *E*_1_,

(24) m(r,f(k+1)f)  ≤m(r,f(k+1)f(k))+m(r,f(k)f)  =O{exp⁡p−1{(σ+ε)log⁡qr}}.

Using the same proof of Lemma  3.6 in [19], we can easily prove the following lemma.

**Lemma 22**

Let Φ(*r*) be a continuous and positive increasing function, defined for *r* on (0, +*∞*), with [*p*, *q*]-order *σ*_[*p*,*q*]_(Φ) = limsup⁡⁡_*r*→+*∞*_(log⁡⁡_*p*_Φ(*r*)/log⁡⁡_*q*_ *r*). Then for any subset *E* of [0, +*∞*) that has finite linear measure, there exists a sequence {*r*_*m*_}, *r*_*m*_ ∉ *E* such that

(25) $$\begin{array}{c} \sigma_{\left[p,q\right]}\left(\Phi \right)=\underset{r_{m}\to +\infty }{\lim}\frac{\log_{p}\Phi \left(r_{m}\right)}{\log_{q}r_{m}}. \end{array}$$

**Lemma 23**

Let *g*_1_(*z*) and *g*_2_(*z*) be two entire functions of [*p*, *q*]-order, and denote *F* = *g*_1_ *g*_2_. Then

(26) $$\begin{array}{c} \lambda_{\left[p,q\right]}\left(F\right)=\max\left\{\lambda_{\left[p,q\right]}\left(g_{1}\right),\lambda_{\left[p,q\right]}\left(g_{2}\right)\right\}. \end{array}$$

**Proof**

Let *n*(*r*, *F*) denote the number of the zeros of *E*(*z*) in disk = {*z* : |*z* | ≤*r*} and so on for *g*_1_ and *g*_2_. Since, for any given *r* > 0, we have *n*(*r*, *F*) ≥ *n*(*r*, *g*_1_) and *n*(*r*, *F*) ≥ *n*(*r*, *g*_2_), thus by Definition 11, we have

(27) $$\begin{array}{c} \lambda_{\left[p,q\right]}\left(F\right)\geq \max\left\{\lambda_{\left[p,q\right]}\left(g_{1}\right),\lambda_{\left[p,q\right]}\left(g_{2}\right)\right\}. \end{array}$$

On the other hand, since the zero of *E*(*z*) must be the zero of *g*_1_ or *g*_2_, then for any given *r* > 0, we have

(28) $$\begin{array}{c} n\left(r,F\right)\leq n\left(r,g_{1}\right)+n\left(r,g_{2}\right)\leq 2\max\left\{n\left(r,g_{1}\right),n\left(r,g_{2}\right)\right\}. \end{array}$$

Therefore, by Definition 11, we have

(29) $$\begin{array}{c} \lambda_{\left[p,q\right]}\left(F\right)\leq \max\left\{\lambda_{\left[p,q\right]}\left(g_{1}\right),\lambda_{\left[p,q\right]}\left(g_{2}\right)\right\}. \end{array}$$

Thus we complete the proof of Lemma 22.

**Lemma 24**

A meromorphic function *f*(*z*) with [*p*, *q*] index can be represented by the form

(30) $$\begin{array}{c} f\left(z\right)=\frac{U\left(z\right)e^{g(z)}}{V\left(z\right)}, \end{array}$$

where *U*(*z*), *V*(*z*), and *g*(*z*) are entire functions such that

(31) $$\begin{array}{c} \lambda_{\left[p,q\right]}\left(f\right)=\lambda_{\left[p,q\right]}\left(U\right)=\sigma_{\left[p,q\right]}\left(U\right),\\ \lambda_{\left[p,q\right]}\left(\frac{1}{f}\right)=\lambda_{\left[p,q\right]}\left(V\right)=\sigma_{\left[p,q\right]}\left(V\right),\\ \sigma_{\left[p,q\right]}\left(f\right)=\max\left\{\sigma_{\left[p,q\right]}\left(U\right),\sigma_{\left[p,q\right]}\left(V\right),\sigma_{\left[p,q\right]}\left(e^{g}\right)\right\}. \end{array}$$

**Proof**

By using the essential part of the factorization theorem for meromorphic function of finite [*p*, *q*]-order and similar to the proof of Lemma  1.8 in [9], we can get the conclusions of this lemma easily.

**Lemma 25 (see [14, Theorem 6.2])**

Let *f* be a meromorphic solution of

(32) $$\begin{array}{c} f^{\left(k\right)}+A_{k-1}\left(z\right)f^{\left(k-1\right)}+\cdots +A_{1}\left(z\right)f^{\prime }+A_{0}\left(z\right)f=0, \end{array}$$

where *A*_0_,…, *A*_*k*−1_ are meromorphic functions in the plane *ℂ*. Assume that not all coefficients *A*_*j*_ are constants. Given a real constant *γ* > 1, and denoting *T*(*r*)∶ = ∑_*j*−0_^*k*−1^ *T*(*r*, *A*_*j*_), one has

(33) $$\begin{array}{c} \log m\left(r,f\right)<T\left(r\right){\left\{\left(\log r\right)\log T\left(r\right)\right\}}^{\gamma },\;if\;\;t=0,\\ \log m\left(r,f\right)<r^{2t+\gamma -1}T\left(r\right){\left\{\log T\left(r\right)\right\}}^{\gamma },\;if\;\;t=0, \end{array}$$

outside of an exceptional set *E*_*t*_ with ∫_*E*_*t*__ *l* ^*t*−1^ *dl* < +*∞*.

**Remark 26**

From the above lemma, we can see that *t* = 1 corresponds to Euclidean measure and *t* = 0 to logarithmic measure.

Using the above lemma, we can get the following lemma.

**Lemma 27**

Let *A*_0_, *A*_1_,…, *A*_*k*−1_ be meromorphic functions with [*p*, *q*] index and 0 ≤ *σ*_[*p*,*q*]_(*A*_*j*_) < *∞*  (*j* = 0,1,…, *k* − 1). If *f* is a meromorphic solution of (20) whose poles are of uniformly bounded multiplicities or *δ*(*∞*, *f*) > 0, then *σ*_[*p*+1,*q*]_(*f*) ≤ max⁡⁡{*σ*_[*p*,*q*]_(*A*_*j*_) : *j* = 0,1,…, *k* − 1}.

**Proof**

Firstly, suppose that *σ*_[*p*,*q*]_(*f*) < *∞*; then we have *σ*_[*p*+1,*q*]_(*f*) = 0. Since 0 ≤ *σ*_[*p*,*q*]_(*A*_*j*_) < *∞*  (*j* = 0,1,…, *k* − 1), then we have *σ*_[*p*+1,*q*]_(*f*) ≤ max⁡{*σ*_[*p*,*q*]_(*A*_*j*_) : *j* = 0,1,…, *k* − 1}. Second, suppose that *σ*_[*p*,*q*]_(*f*) = *∞*. From (32), we know that the poles of *f*(*z*) can only occur at the poles of *A*_0_, *A*_1_,…, *A*_*k*−1_. Since the multiplicities of poles of *f* are uniformly bounded, we have

(34) N(r,f)≤K1N¯(r,f)≤K1∑j=0k−1N¯(r,Aj)≤K0max⁡⁡{N(r,Aj):j=0,1,…,k−1},

where *K*_0_, *K*_1_ are some suitable positive constants. Thus, we can get

(35) T(r,f) =m(r,f)+O(max⁡⁡{N(r,Aj):j=0,1,…,k−1}).

Set *δ*(*∞*, *f*)∶ = *α*_1_ > 0; for sufficiently large *r*, we have

(36) $$\begin{array}{c} m\left(r,f\right)\geq \frac{\alpha_{1}}{2}T\left(r,f\right). \end{array}$$

From Lemma 24 and (35) or (36), we obtain

(37) log⁡⁡T(r,f)≤log⁡⁡m(r,f)+O(log⁡⁡T(r))≤O{T(r){(log⁡⁡r)log⁡T(r)}γ}

or

(38) $$\begin{array}{c} \log T\left(r,f\right)\leq \left(\frac{2}{\alpha_{1}}m\left(r,f\right)\right)\leq O\left\{T\left(r\right){\left\{\left(\log r\right)\log T\left(r\right)\right\}}^{\gamma }\right\} \end{array}$$

outside of an exceptional set *E*_0_ with finite logarithmic measure.

Using a standard method to deal with the finite logarithmic measure set, one immediately gets from the previous inequalities that *σ*_[*p*+1,*q*]_(*f*) ≤ max⁡⁡{*σ*_[*p*,*q*]_(*A*_*j*_) : *j* = 0,1,…, *k* − 1}.

Thus, we complete the proof of this lemma.

**Lemma 28**

Let *A* be a meromorphic function with [*p*, *q*] index, and let *f* be a nonzero meromorphic solution of (1). Then

- if either *δ*(*∞*, *f*) > 0 or the poles of *f* are of uniformly bounded multiplicities, then *σ*_[*p*+1,*q*]_(*f*) ≤ *σ*_[*p*,*q*]_(*A*);
- if *δ*(*∞*, *A*) > 0 or *λ*_[*p*,*q*]_(1/*A*) < *σ*_[*p*,*q*]_(*A*), then *σ*_[*p*+1,*q*]_(*f*) ≥ *σ*_[*p*,*q*]_(*A*).

**Proof**

Suppose that *f* is a nonzero meromorphic solution of (1). It is easy to see that (i) holds since (i) is just a special case of Lemma 25.

Next, we assume that *A* satisfies *δ*(*∞*, *A*) > 0 or *λ*_[*p*,*q*]_(1/*A*) < *σ*_[*p*,*q*]_(*A*). By (1), we have

(39) $$\begin{array}{c} -A\left(z\right)=\frac{f^{\prime \prime }}{f}. \end{array}$$

By (39) and Lemma 21, we can get that

(40) $$\begin{array}{c} m\left(r,A\right)\leq m\left(r,\frac{f^{\prime \prime }}{f}\right)=O\left\{\log\left(rT\left(r,f\right)\right)\right\} \end{array}$$

holds for all sufficiently large *r* ∉ *E*, where *E* ⊂ (0, +*∞*) has finite linear measure. Hence

(41) T(r,A)=m(r,A)+N(r,A)≤N(r,A)+O{log⁡⁡(rT(r,f))}

holds for all sufficiently large |*z* | = *r* ∉ *E*.

If *σ*_[*p*,*q*]_(*A*) = 0, since *A* is a meromorphic function with [*p*, *q*] index, by Definition 6 and Lemma 22, there exists a sequence {*r*_*m*_} such that, for all *r*_*m*_ ∉ *E*_3_,

(42) $$\begin{array}{c} T\left(r_{m},A\right)\geq \exp_{p-1}\left\{M\log_{q}r_{m}\right\} \end{array}$$

holds for any sufficiently large constant *M* > 0. If *σ*_[*p*,*q*]_(*A*) = *σ* > 0, by Lemma 22 there exists a sequence {*r*_*m*_} such that, for all *r*_*m*_ ∉ *E*_3_,

(43) $$\begin{array}{c} T\left(r_{m},A\right)\geq \exp_{p}\left\{\left(\sigma -\varepsilon \right)\log_{q}r_{m}\right\} \end{array}$$

holds for any given *ε*  (0 < *ε* < *σ*).

We will consider two cases as follows.

*Case  1*. Suppose that *δ*(*∞*, *A*)∶ = *α*_2_ > 0. Then for sufficiently large *r*, we have

(44) $$\begin{array}{c} \frac{\alpha_{2}}{2}T\left(r,A\right)\leq m\left(r,A\right)=O\left\{\log\left(rT\left(r,f\right)\right)\right\}. \end{array}$$

If *σ*_[*p*,*q*]_(*A*) = 0 and *A* has [*p*, *q*] index, from (42) and (44), we have *σ*_[*p*+1,*q*]_(*f*) ≥ 0 = *σ*_[*p*,*q*]_(*A*). If *σ*_[*p*,*q*]_(*A*) > 0, then from (43) and (44), we can get that *σ*_[*p*+1,*q*]_(*f*) ≥ *σ*_[*p*,*q*]_(*A*).

*Case  2*. Suppose that *λ*_[*p*,*q*]_(1/*A*) < *σ*_[*p*,*q*]_(*A*) = *σ*. Then the inequality

(45) $$\begin{array}{c} N\left(r,A\right)\leq \exp_{p}\left\{\left(\lambda_{\left[p,q\right]}\frac{1}{A}+\varepsilon \right)\log_{q}r\right\} \end{array}$$

holds for any given *ε*(0 < 2*ε* < *σ* − *λ*_[*p*,*q*]_(1/*A*)). Then from (41), (43), and (45), we can get that *σ*_[*p*+1,*q*]_(*f*) ≥ *σ*_[*p*,*q*]_(*A*).

Thus, we complete the proof of Lemma 28.

## 3. Proof of Theorem 13

Since *σ*_[*p*,*q*]_(*A*)∶ = *σ* > 0 and *f* is a solution of (1), it is easy to see that *f* can not be rational nor be of the form *e* ^*az*+*b*^ for constants *a* and *b*. Thus, by Lemma 19 we have

(46) T(r,ff′)=O(N¯(r,f)+N¯(r,1f)+N¯(r,1f′′)),n.e.  as  r→∞.

From (1), we have

(47) $$\begin{array}{c} \bar{N}\left(r,\frac{1}{f^{\prime \prime }}\right)\leq \bar{N}\left(r,\frac{1}{f}\right)+\bar{N}\left(r,\frac{1}{A}\right). \end{array}$$

Suppose that (9) fails to hold that is

(48) $$\begin{array}{c} \sigma_{\left[p,q\right]}\left(A\right)>\max\left\{{\bar{\lambda }}_{\left[p,q\right]}\left(f\right),{\bar{\lambda }}_{\left[p,q\right]}\left(\frac{1}{f}\right)\right\}; \end{array}$$

by the assumption ${\bar{\lambda }}_{\left[p,q\right]}(A)<\sigma_{\left[p,q\right]}(A)$ and from (46)–(48), we can get *σ*_[*p*,*q*]_(*f*/*f*′) < *σ*_[*p*,*q*]_(*A*).

Set *ψ* = *f*′/*f*; by the first main theorem, we can get that

(49) $$\begin{array}{c} \sigma_{\left[p,q\right]}\left(\psi \right)<\sigma_{\left[p,q\right]}\left(A\right). \end{array}$$

From (1) and *ψ* = *f*′/*f*, we can get −*A* = *ψ*′ + *ψ* ^2^. Thus, from (49) and Lemma 20, we have *σ*_[*p*,*q*]_(*A*) ≤ *σ*_[*p*,*q*]_(*ψ*) < *σ*_[*p*,*q*]_(*A*), a contradiction. Thus, (9) is true.

In the special case where either *δ*(*∞*, *f*) > 0 or the poles of *f* are of uniformly bounded multiplicities, by Lemma 28 we have

(50) $$\begin{array}{c} \max\left\{\lambda_{\left[p+1,q\right]}\left(\frac{1}{f}\right),\lambda_{\left[p+1,q\right]}\left(f\right)\right\}\leq \sigma_{\left[p+1,q\right]}\left(f\right)\leq \sigma_{\left[p,q\right]}\left(A\right). \end{array}$$

Combining the above discussions, we can get (10).

Thus, this completes the proof of Theorem 13.

## 4. Proof of Theorem 14

Suppose that (1) possesses two linearly independent meromorphic solutions *f*_1_ and *f*_2_ such that ${\bar{\lambda }}_{\left[p,q\right]}(E)<\infty$, where *E*_1_∶ = *E* = *f*_1_ *f*_2_. Let *f* = *af*_1_ + *bf*_2_, where *a* and *b* are nonzero constants, and set *E*_2_∶ = *ff*_1_. From (1), we can see that any pole of *f* is a pole of *A*. Since ${\bar{\lambda }}_{\left[p,q\right]}\left(1/A\right)\leq \sigma_{\left[p,q\right]}(A)$, and by the assumptions of Theorem 14, we have ${\bar{\lambda }}_{\left[p,q\right]}\left(1/f\right)\leq {\bar{\lambda }}_{\left[p,q\right]}\left(1/A\right)<\infty$. If ${\bar{\lambda }}_{\left[p,q\right]}(f)<\infty$, from the above discussion, we can get that ${\bar{\lambda }}_{\left[p,q\right]}(E_{1})<\infty$ and ${\bar{\lambda }}_{\left[p,q\right]}(E_{2})<\infty$. By Lemma D(e) in [6], there exists a constant *c* > 0 such that n.e. as *r* → *∞*,

(51) T(r,Ej)=O(N¯(r,1Ej)+T(r,A)+log⁡r)=O(exp⁡p(clog⁡qr)+T(r,A))

for *j* = 1,2. Since *E*_2_ = *af*_1_^2^ + *bE*_1_, from (51) we can get that n.e. as *r* → *∞*,

(52) $$\begin{array}{c} T\left(r,f_{1}\right)=O\left(\exp_{p}\left(c\log_{q}r\right)+T\left(r,A\right)\right). \end{array}$$

From *A* = −(*f*′′/*f*) and Lemma 21, we can get

(53) $$\begin{array}{c} m\left(r,A\right)=O\left(\log\left(rT\left(r,f\right)\right)\right),\;\text{n}.\text{e}.\;\;r\to \infty . \end{array}$$

And from (1), we can see that any pole of *A* is at most double and is either a zero or pole of *f*. Then we get

(54) $$\begin{array}{c} N\left(r,A\right)\leq 2\left(\bar{N}\left(r,\frac{1}{f}\right)+\bar{N}\left(r,f\right)\right). \end{array}$$

By assumptions ${\bar{\lambda }}_{\left[p,q\right]}(f)<\infty$, ${\bar{\lambda }}_{\left[p,q\right]}\left(1/f\right)<\infty$ and (54), we can get that *N*(*r*, *A*) = *O*(exp⁡_*p*_(*d*log⁡⁡_*q*_ *r*)) as *r* → *∞* for some *d* > 0. Together with (52), (53), and (54), we can get that *T*(*r*, *f*_1_) = *O*(exp⁡_*p*_(*d* log⁡⁡_*q*_ *r*)) n.e. as *r* → *∞*. Thus, it follows that *f*_1_ is of finite [*p*, *q*]-order.

By the identity of Abel, we have

(55) $$\begin{array}{c} {\left(\frac{f_{2}}{f_{1}}\right)}^{\prime }=\frac{W}{f_{1}^{2}}, \end{array}$$

where *W* is equal to the Wronskian of *f*_1_ and *f*_2_. Hence, by Lemma 20 and (55), we get

(56) σ[p,q](f2)=σ[p,q](f1f2f1)≤max⁡⁡{σ[p,q](f2f1),σ[p,q](f1)}=σ[p,q](f1).

Reversing the roles of *f*_1_ and *f*_2_, we can get that *σ*_[*p*,*q*]_(*f*_1_) = *σ*_[*p*,*q*]_(*f*_2_). Thus, we can get that all solutions of (1) are of finite [*p*, *q*]-order if ${\bar{\lambda }}_{\left[p,q\right]}(f)<\infty$.

In special case where *δ*(*∞*, *A*) > 0 or *λ*_[*p*,*q*]_(1/*A*) < *σ*_[*p*,*q*]_(*A*), by Lemma 28, we can get that all meromorphic solutions *f*≢0 of (1) satisfy *σ*_[*p*+1,*q*]_(*f*) ≥ *σ* = *σ*_[*p*,*q*]_(*A*). Hence, we can obtain that ${\bar{\lambda }}_{\left[p,q\right]}(f)=\infty$ holds for any solution *f*≢0 of (1) which is not a constant multiple of either *f*_1_ or *f*_2_.

## 5. Proof of Theorem 15

From [6, Page 664], we can get *σ*_[*p*+1,*q*]_(*f*_1_) = *σ*_[*p*+1,*q*]_(*f*_2_) easily. Suppose that *δ*(*∞*, *A*)∶ = *α*_3_ > 0 or *λ*_[*p*,*q*]_(1/*A*) < *σ*_[*p*,*q*]_(*A*) and that either *δ*(*∞*, *f*) > 0 or the poles of *f* are of uniformly bounded multiplicities. Then by Lemma 28 we obtain

(57) σ[p+1,q](E)≤max⁡{σ[p+1,q](f1),σ[p+1,q](f2)}≤σ[p+1,q](f1)=σ[p+1,q](f2)=σ[p,q](A)<∞.

By Lemma D(e) in [6], there a constant *c* > 0 such that n.e. as *r* → *∞*,

(58) T(r,E)=O(N¯(r,1E)+T(r,A)+log⁡r)=O(exp⁡p(c log⁡⁡qr)+T(r,A)).

By Lemmas 21 and 28, we have

(59) m(r,A)=m(r,f1′′f1)=O(log⁡⁡(rT(r,f1)))=O(exp⁡p(β1log⁡⁡qr))

for some *β*_1_ < *∞* outside of a possible exceptional set *E*_4_ ⊂ [0, *∞*) with finite linear measure. If *δ*(*∞*, *A*)∶ = *α*_3_ > 0, then for sufficiently large *r*, we have

(60) α32T(r,A)≤m(r,A)=O(exp⁡p(β1log⁡⁡qr)), r∉E4.

If *λ*_[*p*,*q*]_(1/*A*) < *σ*_[*p*,*q*]_(*A*) < *∞*, there exists a constant *β*_2_ < *∞* such that

(61) $$\begin{array}{c} N\left(r,A\right)=O\left(\exp_{p}\left(\beta_{2}\log_{q}r\right)\right). \end{array}$$

From the above equality and (60), we have

(62) T(r,A)=m(r,A)+N(r,A)=O(exp⁡⁡p(β log⁡⁡qr)),

where *β* = max⁡⁡{*β*_1_, *β*_2_}.

Therefore, together with (58) and either (60) or (62), we obtain

(63) $$\begin{array}{c} T\left(r,E\right)=O\left(\bar{N}\left(r,\frac{1}{E}\right)+\exp_{p}\left(\beta {\;\log}_{q}r\right)\right),\;r\notin E_{4}. \end{array}$$

Suppose that ${\bar{\lambda }}_{\left[p+1,q\right]}(E)<\sigma_{\left[p+1,q\right]}(E)∶=\sigma_{1}$; then from Definitions 6 and 11 we have $\bar{N}\left(r,1/E\right)=O\left(\exp_{p+1}\left(b\log_{q}r\right)\right)$ for some *b* < *σ*_1_. From (63), *T*(*r*, *E*) = *O*(exp⁡_*p*+1_(*b* log⁡⁡_*q*_ *r*)), *r* ∉ *E*_4_, and then by standard reasoning, we obtain *σ*_[*p*+1,*q*]_(*E*) ≤ *b* < *σ*_1_ = *σ*_[*p*+1,*q*]_(*E*). Thus, we get a contradiction. Therefore, we have ${\bar{\lambda }}_{\left[p+1,q\right]}(E)\geq \sigma_{1}=\sigma_{\left[p+1,q\right]}(E)$. And since $\sigma_{\left[p+1,q\right]}(E)\geq \lambda_{\left[p+1,q\right]}(E)\geq {\bar{\lambda }}_{\left[p+1,q\right]}(E)$, then we have $\sigma_{\left[p+1,q\right]}(E)=\lambda_{\left[p+1,q\right]}(E)={\bar{\lambda }}_{\left[p+1,q\right]}(E)$.

By Lemma D(a) in [6], *f*_1_ and *f*_2_ have no common zeros. Let *f*_*j*_ = (*g*_*j*_/*d*_*j*_)  (*j* = 1,2), where *g*_*j*_ and *d*_*j*_ have no common zeros. This implies that *g*_1_ and *g*_2_ have no common zeros, that *λ*_[*p*,*q*]_(*f*_*j*_) = *λ*_[*p*,*q*]_(*g*_*j*_) for *j* = 1,2, and that *λ*_[*p*,*q*]_(*E*) = *λ*_[*p*,*q*]_(*g*_1_ *g*_2_). Then by Lemma 23, we have *λ*_[*p*+1,*q*]_(*E*) = max⁡{*λ*_[*p*+1,*q*]_(*f*_1_), *λ*_[*p*+1,*q*]_(*f*_2_)}.

Thus, we can get the following conclusion

(64) λ¯[p+1,q](E)=λ[p+1,q](E)=σ[p+1,q](E)=max⁡{λ¯[p+1,q](f1),λ¯[p+1,q](f2)}≤λ[p+1,q](f1)=λ[p+1,q](f2)=λ[p,q](A)<∞.

Therefore, we complete the proof of Theorem 15.

## 6. Proof of Theorem 16

From *E*∶ = *f*_1_ *f*_2_ and *F*∶ = *g*_1_ *g*_2_, by using a similar argument as in [9, Lemma  1.7], we can get *λ*_[*p*,*q*]_(*F*) = max⁡⁡{*λ*_[*p*,*q*]_(*g*_1_), *λ*_[*p*,*q*]_(*g*_1_)}. Suppose that *λ*_[*p*,*q*]_(*F*) < *λ*_[*p*,*q*]_(*A*)∶ = *σ*_1_ from (12), we have

(65) $$\begin{array}{c} \bar{N}\left(r,\frac{1}{E}\right)=O\left(\exp_{p}\left(\beta_{1}\log_{q}r\right)\right) \end{array}$$

for some *β*_1_ < *σ*_[*p*,*q*]_(*A*) = *σ*_1_. Since *λ*_[*p*,*q*]_(*A*)∶ = *σ*_1_ > 0 and from Definition 6, for any *ε*  (>0), we have

(66) $$\begin{array}{c} T\left(r,A\right)=O\left\{\exp_{p}\left(\left(\sigma_{1}+\varepsilon \right)\log_{q}r\right)\right\}. \end{array}$$

By Lemma D(e) in [6], we have $T(r,E)=O\left(\bar{N}\left(r,1/E\right)+T(r,A)+\log r\right)$. Thus, we can get that

(67) $$\begin{array}{c} T\left(r,E\right)=O\left\{\exp_{p}\left(\left(\sigma_{1}+\varepsilon \right)\log_{q}r\right)\right\}. \end{array}$$

Hence, from the above equality, we have *σ*_[*p*,*q*]_(*E*) ≤ *σ*_1_. On the other hand, by Lemma B(iv) in [6], we have

(68) $$\begin{array}{c} 4A={\left(\frac{E^{\prime }}{E}\right)}^{2}-2\frac{E^{\prime \prime }}{E}-\frac{1}{E^{2}}, \end{array}$$

which implies that *σ*_1_ ≤ *σ*_[*p*,*q*]_(*E*). Since *σ*_[*p*,*q*]_(Π) < *σ*_[*p*,*q*]_(*A*), using the same argument as in the above for the function *F*, we have *σ*_[*p*,*q*]_(*E*) = *σ*_[*p*,*q*]_(*F*) = *σ*_[*p*,*q*]_(*A*) = *σ*_1_.

From the assumptions of Theorem 16 and Lemma 24, we can write

(69) $$\begin{array}{c} E=\frac{U_{1}e^{G_{1}}}{V_{1}},\;\;F=\frac{U_{2}e^{G_{2}}}{V_{2}}, \end{array}$$

where *σ*_[*p*,*q*]_(*U*_1_) = *λ*_[*p*,*q*]_(*E*) < *σ*_[*p*,*q*]_(*A*) and *σ*_[*p*,*q*]_(*U*_2_) = *λ*_[*p*,*q*]_(*F*) < *σ*_[*p*,*q*]_(*A*). And since max⁡{*λ*_[*p*,*q*]_(1/*E*), *λ*_[*p*,*q*]_(1/*F*)} < *λ*_[*p*,*q*]_(*A*), we have

(70) $$\begin{array}{c} \sigma_{\left[p,q\right]}\left(e^{G_{1}}\right)=\sigma_{\left[p,q\right]}\left(e^{G_{2}}\right)=\sigma_{\left[p,q\right]}\left(A\right). \end{array}$$

Substituting (69) into (68), and using the same argument as in the proof of Theorem  3.1 in [7], we can get

(71) $$\begin{array}{c} ce^{2(G_{1}-G_{2})}=-\frac{V_{1}^{2}U_{2}^{2}}{U_{1}^{2}V_{2}^{2}}, \end{array}$$

where *c* ≠ 0. From (68), we have

(72) $$\begin{array}{c} \frac{E^{2}}{F^{2}}=\frac{U_{1}^{2}V_{2}^{2}}{U_{2}^{2}V_{1}^{2}}e^{2(G_{1}-G_{2})}=-\frac{1}{c}. \end{array}$$

For the function *F*, similar to (68), we have

(73) $$\begin{array}{c} 4\left(A+\Pi \right)={\left(\frac{F^{\prime }}{F}\right)}^{2}-2\frac{F^{\prime \prime }}{F}-\frac{1}{F^{2}}. \end{array}$$

From (68), (72), and (73), we have

(74) $$\begin{array}{c} 4\left(A+\Pi +\frac{1}{c}A\right)={\left(\frac{F^{\prime }}{F}\right)}^{2}-2\frac{F^{\prime \prime }}{F}+\frac{1}{c}{\left(\frac{E^{\prime }}{E}\right)}^{2}-\frac{2}{c}\frac{E^{\prime \prime }}{E}. \end{array}$$

Since *σ*_[*p*,*q*]_(*E*) = *σ*_[*p*,*q*]_(*F*) = *σ*_[*p*,*q*]_(*A*) = *σ*_1_, by Lemma 22, for any *ε*  (>0), we can get

(75) T(r,A(1+1c)+Π) =m(r,A(1+1c)+Π)+N(r,A(1+1c)+Π) =O(exp⁡p−1{(σ1+ε)log⁡⁡qr}) n.e.  as  r→∞.

From (75), we can get *σ*_[*p*,*q*]_(*A*(1 + (1/*c*)) + Π) = 0 < *σ*_1_ = *σ*_[*p*,*q*]_(*A*) easily. Thus, we can get *c* = −1. Since *E* ^2^ = *F* ^2^, we have

(76) $$\begin{array}{c} \frac{E^{\prime }}{E}=\frac{F^{\prime }}{F},\;\;\frac{E^{\prime \prime }}{E}=\frac{F^{\prime \prime }}{F}. \end{array}$$

Then from (68) and (73), we have Π ≡ 0, a contradiction.

Therefore, we complete the proof of Theorem 16.
